# Cardiac Amyloidosis Disguised as Atrial Flutter: A Case Report

**DOI:** 10.7759/cureus.39524

**Published:** 2023-05-26

**Authors:** Guarina Molina, Jahangir Rouzbehani Selakhor, Melissa Alvarez, Rafael Contreras, Uneza R Khawaja

**Affiliations:** 1 Internal Medicine, Danbury Hospital, Danbury, USA; 2 Cardiology, Danbury Hospital, Danbury, USA

**Keywords:** pyp scintigraphy, heart failure, echocardiogram, cmr, attr, cardiac amyloid, arrhythmia, atrial flutter

## Abstract

Cardiac amyloidosis (CA) is a rare form of infiltrative cardiomyopathy (IC) that frequently leads to heart failure (HF). Its symptoms can range from minimal to significant shortness of breath, palpitations, leg swelling, and chest discomfort. Early diagnosis and treatment are crucial in preventing the further progression of the disease and improving outcomes. This case report describes a 63-year-old male with no prior medical history who presented with severe dyspnea, palpitations, and chest heaviness. Initially diagnosed with atrial flutter, he was later confirmed to have cardiac amyloidosis through a thorough workup with multimodality imaging. The patient was started on guideline-directed medical therapy (GDMT) and discharged home with a follow-up from a heart failure specialist. An outpatient workup confirmed the diagnosis of amyloidosis with a positive pyrophosphate scan. At a seven-month follow-up, the workup for extra-cardiac involvement was negative, and the ejection fraction (EF) had improved. This case highlights the importance of a high index of suspicion and a thorough workup in cases of suspected cardiac amyloidosis to achieve early diagnosis and prevent disease progression.

## Introduction

Cardiac amyloidosis (CA) is a rare form of infiltrative cardiomyopathy (IC) caused by amyloid deposition in the extracellular space of the heart and a frequently undiagnosed cause of heart failure (HF). Breakthroughs in non-invasive testing and medical therapy have increased awareness and clinical suspicion, leading to earlier diagnosis and decreased progression. This case demonstrates the course of a patient initially diagnosed with atrial flutter who, after a thorough workup with multimodality imaging, was later confirmed to have cardiac amyloidosis.

## Case presentation

This is a 63-year-old male with no past medical history who presented to the emergency department with severe dyspnea, palpitations, and chest heaviness that started a few hours prior. His family and social history were noncontributory, while the surgical history was remarkable for bilateral carpal tunnel release six years before.

**Figure 1:**
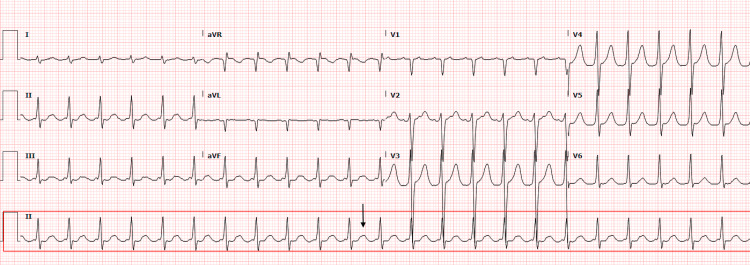
Electrocardiogram (EKG) from admission EKG from admission showing atrial flutter with a rapid ventricular response (141 bpm) appreciated in the rhythm strip (II) and flutter rhythm signalized with an arrow bpm, beats per minute; aVR, augmented vector right; aVL, augmented vector left; aVF, augmented vector foot

At presentation, he was afebrile, his heart rate (HR) was 141 beats per minute (bpm), his blood pressure was 149/118 mmHg with oxygen saturation of 100% on room air. Physical examination was unremarkable, with no cardiopulmonary findings. Pertinent bloodwork included N-terminal pro-B-type natriuretic peptide (NT-proBNP) of 3,491 pg/mL (reference: <125 pg/mL), high-sensitivity fifth-generation troponin T (hs-TnT) of 39 ng/L (reference: <14 ng/L), and creatinine level of 1.64 mg/dL. The electrocardiogram (EKG) showed a 2:1 atrial flutter with a rapid ventricular response with borderline right axis deviation but no ST or T wave abnormalities (Figure 1). Initial management included oral metoprolol tartrate 25 mg twice a day and admission to the cardiology service for further monitoring.

In the first 24 hours, he was monitored on telemetry in anticipation of spontaneous conversion to sinus rhythm and started on anticoagulation with a therapeutic dose of enoxaparin. Transthoracic echocardiogram (TTE) showed severe concentric left ventricular (LV) hypertrophy with ventricular septum of 1.7 cm (reference: 0.6-1.0 cm), an ejection fraction (EF) of 20%, and bright and speckled appearance of the myocardium, raising suspicion for IC. The following day, he underwent transesophageal echocardiogram (TEE) with direct current cardioversion, resulting in the successful restoration of sinus rhythm. A few hours later, he was noted to have isolated 12-second runs of non-sustained ventricular tachycardia and episodes of atrial tachycardia without recurrence.

**Figure 2:**
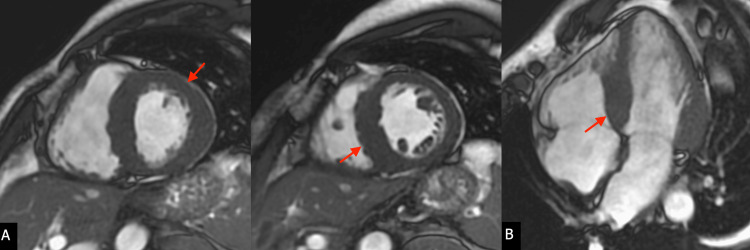
Cine SSFP views of the LV showing severe hypertrophy (A) Short axis view and (B) four-chamber view SSFP, steady-state free precession; LV, left ventricle

**Figure 3:**
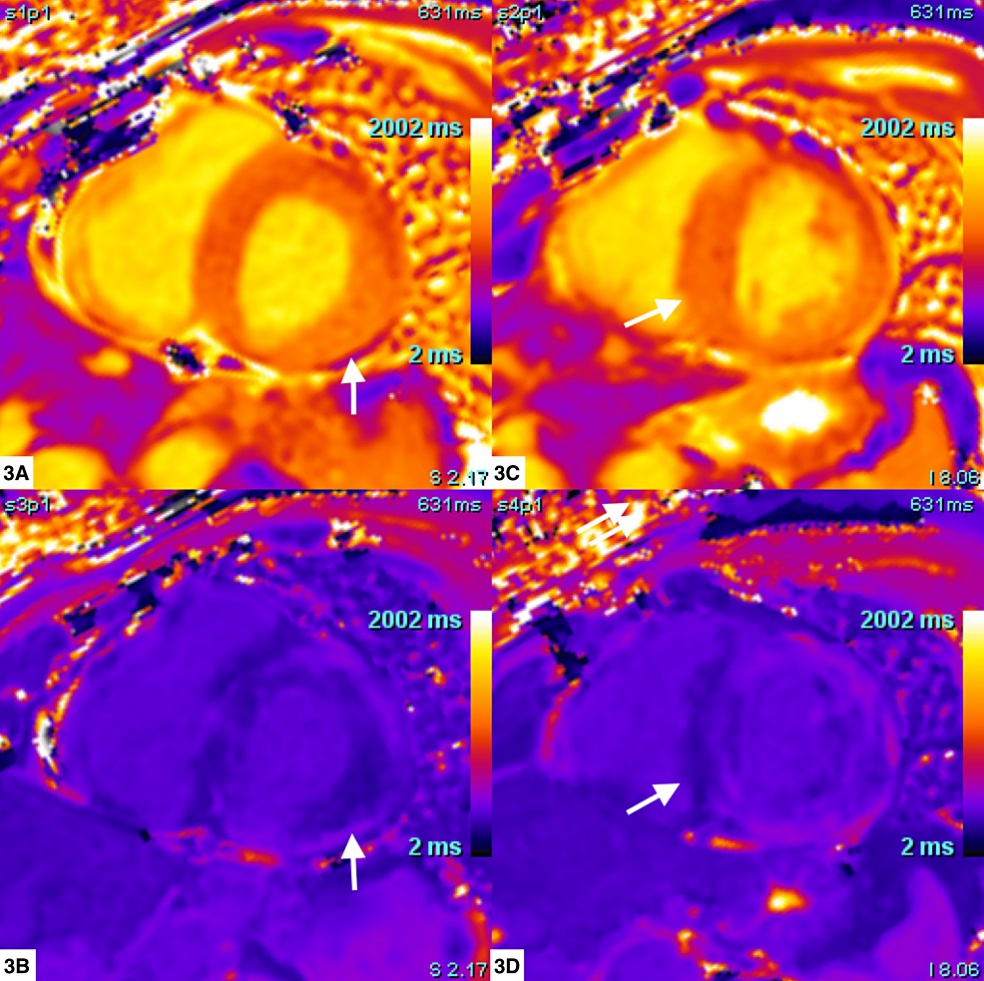
Native T1 maps in short axis view The native T1 relaxation time is diffusely elevated at the basal (3A, pre-contrast; 3B, post-contrast) to mid-myocardial (3C, pre-contrast; 3D, post-contrast) segments

**Figure 4:**
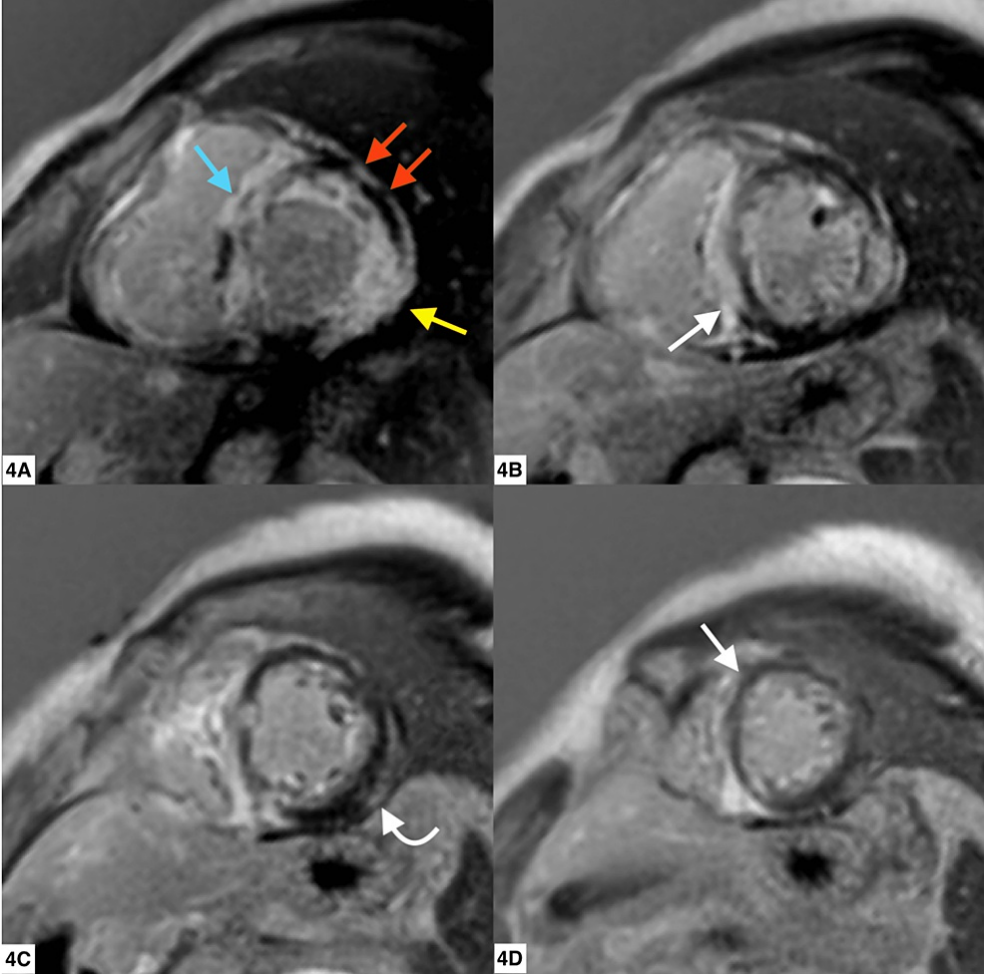
Short axis views with delayed gadolinium enhancement (DGE) On DGE, there is an extensive enhancement of the basal (4A, blue arrow) to apical (4D) septum and basal anterior (4A, red arrows), basal (4A, yellow arrow) to mid-lateral (4C), and basal inferior walls (4B)

Cardiovascular magnetic resonance (CMR) was performed on a 1.5 Tesla Artis unit (Siemens Medical Solutions USA, Inc., Malvern, PA) using balanced steady-state imaging/bright blood (Figure 2A-2B). T1 mapping (Figure 3), T2-weighted triple inversion, and gadolinium-based contrast administration (Figure 4) were used, showing the enhancement of the right ventricular myocardium and of the septum and basal walls of the LV: transmural (basal segments) and subendocardial patterns (mid-segments). Due to concerns for cardiac amyloidosis, additional immunology workup was ordered, with unremarkable results (Table 1).

**Table 1:** Testing for light chain amyloid markers IFE, immunofixation; IgG, immunoglobulin G; MGUS, monoclonal gammopathy of undetermined significance

Marker	Result	Reference Range	Unit
Albumin fraction	3.7	3.7-5.5	g/dL
Alpha-1 globulin	0.15	0.05-0.22	g/dL
Alpha-2 globulin	0.50	0.55-0.87	g/dL
Beta globulin	0.62	0.53-1.11	g/dL
Gamma globulin	0.74	0.41-1.18	g/dL
Protein electrophoresis interpretation	Normal protein distribution by electrophoresis; no abnormal discrete bands suggestive of monoclonal gammopathy		
IFE interpretation	Faint IgG lambda monoclonal band consistent with MGUS		
Urine protein electrophoresis interpretation	Possible abnormal discrete band suspicious for an M-protein observed in the beta region of protein electrophoresis of neat urine		
Urine IFE interpretation	No monoclonal bands detected in concentrated urine		

Guideline-directed medical therapy (GDMT), including metoprolol succinate 100 mg daily, sacubitril-valsartan 24-26 mg twice a day, spironolactone 25 mg daily, and anticoagulation with apixaban 5 mg twice a day, was started. He was discharged home after 48 hours with follow-up scheduled with HF specialist.

**Figure 5:**
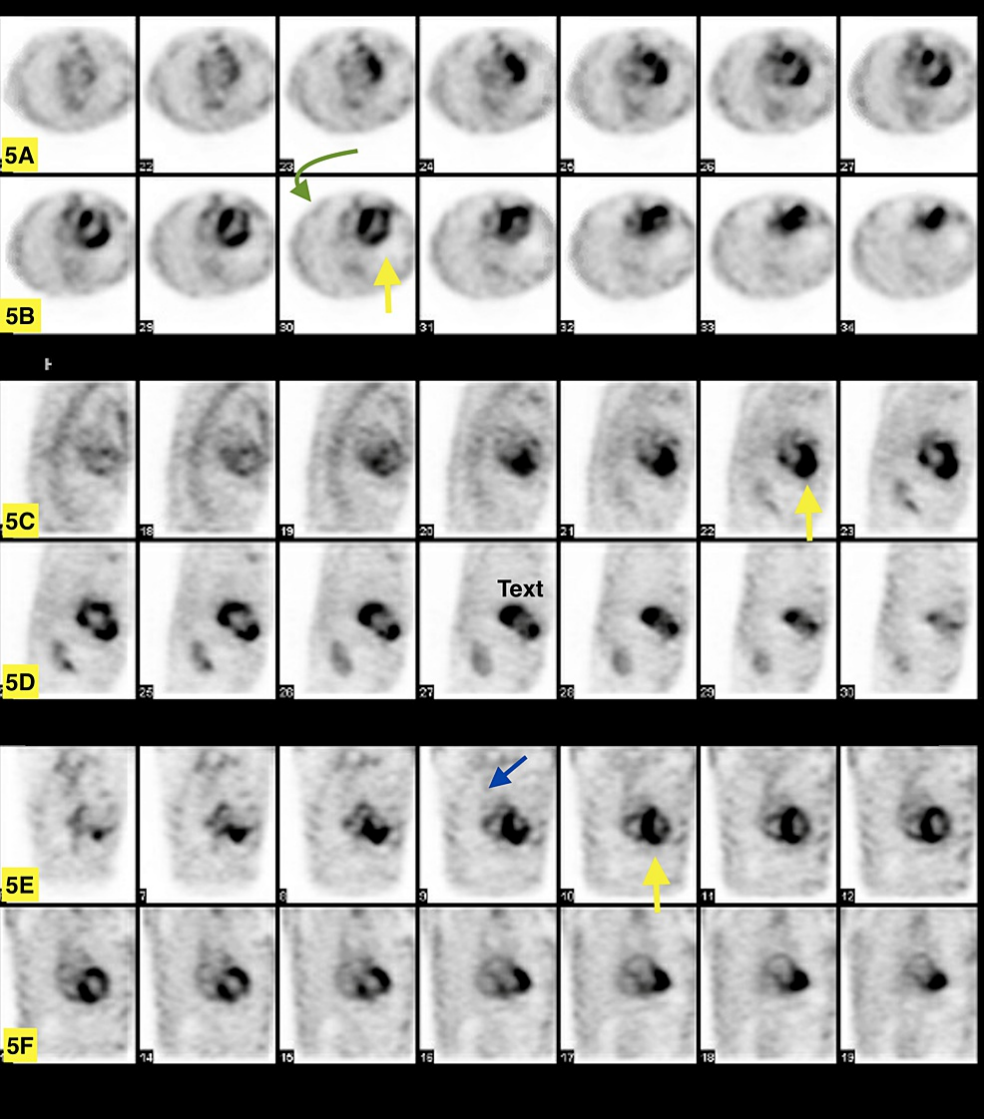
Technetium-99m pyrophosphate (Tc-99m PYP) scintigraph (5A and 5B) Head to feet transversal cut; slice thickness 6.78 mm; the heart darker than (yellow arrow) the ribs (green arrow), signalizing increased uptake. (5C and 5D) Right to left sagittal cuts; slice thickness 6.78 mm; heart signaled with a yellow arrow. (5E and 5F) Anterior to posterior coronal cuts; slice thickness 6.78 mm; heart (yellow arrow) and contralateral lung (blue arrow) signaled

The diagnosis of amyloidosis by a positive pyrophosphate (PYP) scan showing grade 3 uptake at the region of interest in the heart and normal uptake in the bone, where uptake in the heart is greater than that of the bone (Figure 5). The heart-to-contralateral lung (H/CL) ratio was 2.03. After confirmatory testing, GDMT was readjusted with metoprolol succinate decreased to 25 mg daily, spironolactone decreased to 25 mg daily, and sacubitril-valsartan stopped. Tafamidis 61 mg daily was prescribed.

At the seven-month follow-up, the workup for extra-cardiac involvement (Table 2) was negative, and the patient's EF had increased to 58%. A TTE performed after a year later showed an EF of 67%.

**Table 2:** Evaluation results for the renal involvement of amyloid disease *Hepatitis panel: hepatitis B core Ab total; hepatitis B surface Ab QI; hepatitis B surface Ag; hepatitis C Ab Ab, antibody; ANA, anti-nuclear antibody; ds, double stranded; GBM, glomerular basement membrane; IgA, immunoglobulin A; IgG, immunoglobulin G; IgM, immunoglobulin M; MPO, myeloperoxidase

Marker	Result	Reference Range	Unit
Hepatitis panel*	Non-reactive		
Complement C4	31	15-53	mg/dL
Complement component C3c	94	82-185	mg/dL
IgA	286	70-320	mg/dL
IgG	1,156	600-1,540	mg/dL
IgM	123	50-300	mg/dL
ANA screen	Negative		
Chromatin (nucleosomal) Ab	<1.0 (negative)		
DNA (ds) Ab	94	>10 (positive)	IU/mL
GBM Ab (IgG)	<1.0*	<1.0*	AI
MPO	<1.0*	<1.0*	AI
24-hour urine protein	112	<150	mg/24 hours
24-hour urine sodium	120	52-389	mmol/24 hours
24-hour urine creatinine	1.65	0.50-2.15	g/24 hours
Protein/creatinine ratio	0.068	<0.100	
Serum kappa light chain (free)	19.6	3.3-19.4	mg/L
Serum lambda light chain (free)	15.3	5.7-26.3	mg/L
Serum kappa/lambda light chain ratio	1.28	0.26-1.65	
Urine kappa light chain (free)	25.10	<32.9	mg/L
Urine lambda light chain (free)	2.86	<3.79	mg/L
Urine kappa/lambda light chain ratio	2.86		

## Discussion

Cardiac amyloidosis is a type of infiltrative cardiomyopathy caused by amyloid fibril deposition in the extracellular space of the heart and, as a syndrome, is classified between transthyretin amyloidosis (ATTR) and light chain amyloidosis (AL) depending on the precursor of the protein. In ATTR, amyloid fibrils are produced from transthyretin protein produced in the liver and may be associated with age (wild-type transthyretin amyloidosis, ATTRwt) or hereditary (ATTRv), inherited in an autosomal-dominant pattern [1].

Common symptoms include dyspnea, lower extremity edema, ascites, and syncope due to atrioventricular node dysfunction; extra-cardiac involvement may affect the renal (proteinuria and nephrotic syndrome), nervous (paresthesias, weakness, and autonomic dysfunction), and musculoskeletal systems (bilateral carpal tunnel syndrome). Approximately 13% of patients diagnosed with heart failure with preserved ejection fraction (HFpEF), presenting with dyspnea, is associated with a diagnosis of CA [2].

The diagnosis of CA requires a high index of suspicion since it may present with a myriad of symptoms in different organ systems without a specific time frame. Echocardiography is the easiest and most available tool to identify hypertrophy and raise suspicion of CA, with common clues including increased LV wall thickness and diastolic dysfunction, as presented with our patient [2]. These features are present in advanced disease, limiting the findings if performed at early stages.

Other diagnostic tools, such as CMR, provide high-definition imaging, and the use of gadolinium-contrast agents is a strongly sensitive tool for diagnosis, with the enhancement of the LV present in 100% of cases [1]. Despite aiding in diagnosis and allowing for non-invasive monitoring and follow-up, these tools are unable to differentiate between the types of amyloid disease.

Bone scintigraphy (technetium-99m pyrophosphate {Tc-99m PYP}) adds to the diagnostic value of echocardiography and CMR by distinguishing CA from other types of hypertrophic disease. An H/CL uptake of >1.5 at one hour has 100% specificity in distinguishing ATTR from AL. When combined with serum and urine monoclonal protein screening, there is a strong negative predictive value toward AL. Endomyocardial biopsy with Congo red staining remains the gold standard for diagnosis and is still performed in cases with high suspicion of amyloid disease with equivocal findings [1,3].

The management of CA after a confirmed diagnosis relies on managing the symptoms of heart failure (loop diuretics and low-dose beta-blockers), preventing atrial fibrillation and other arrhythmias (rate-controlling agents and systemic anticoagulation), and disease-modifying agents, such as ATTR stabilizers to avoid the progression of disease [1].

## Conclusions

Thorough cardiac workup and high clinical suspicion are paramount for any patient who presents with new-onset cardiac symptoms and subtle clues of underlying disease, as cardiac conditions can debut with nontraditional symptoms. If untreated, CA can be fatal within a few months, and early recognition is key to lower disease burden and mortality. Differential diagnoses of our patient, who presented with elevated NT-proBNP and abnormal TTE findings, included hypertrophic cardiomyopathy, Fabry disease, hemochromatosis, and sarcoidosis, which were ruled out due to the rapid progression of the disease and the patient's characteristics.
